# The immunometabolite S-2-hydroxyglutarate exacerbates perioperative ischemic brain injury and cognitive dysfunction by enhancing CD8^+^ T lymphocyte-mediated neurotoxicity

**DOI:** 10.1186/s12974-022-02537-4

**Published:** 2022-07-07

**Authors:** Faqiang Zhang, Mu Niu, Kaikai Guo, Yulong Ma, Qiang Fu, Yanhong Liu, Zeguo Feng, Weidong Mi, Long Wang

**Affiliations:** 1 Department of Anesthesiology, The First Medical Center, Chinese PLA General Hospital, Beijing, 100853 China; 2 Department of Neurology, The Affiliated Hospital of Xuzhou Medical University, Xuzhou Medical University, Jiangsu, 221002 China; 3 Department of Pain Medicine, The First Medical Center, Chinese PLA General Hospital, Beijing, 100853 China; 4 Department of Anesthesiology, Shanghai Pulmonary Hospital, Tongji University School of Medicine, Shanghai, 200433 China

**Keywords:** Brain injury, Perioperative complication, CD8^+^ T lymphocyte, S-2-Hydroxyglutarate, Neurotoxicity, Perioperative stroke, Ischemic stroke

## Abstract

**Background:**

Metabolic dysregulation and disruption of immune homeostasis have been widely associated with perioperative complications including perioperative ischemic stroke. Although immunometabolite S-2-hydroxyglutarate (S-2HG) is an emerging regulator of immune cells and thus triggers the immune response, it is unclear whether and how S-2HG elicits perioperative ischemic brain injury and exacerbates post-stroke cognitive dysfunction.

**Methods:**

Perioperative ischemic stroke was induced by transient middle cerebral artery occlusion for 60 min in C57BL/6 mice 1 day after ileocecal resection. CD8^+^ T lymphocyte activation and invasion of the cerebrovascular compartment were measured using flow cytometry. Untargeted metabolomic profiling was performed to detect metabolic changes in sorted CD8^+^ T lymphocytes after ischemia. CD8^+^ T lymphocytes were transfected with lentivirus ex vivo to mobilize cell proliferation and differentiation before being transferred into recombination activating gene 1 (Rag1^−/−^) stroke mice.

**Results:**

The perioperative stroke mice exhibit more severe cerebral ischemic injury and neurological dysfunction than the stroke-only mice. CD8^+^ T lymphocyte invasion of brain parenchyma and neurotoxicity augment cerebral ischemic injury in the perioperative stroke mice. CD8^+^ T lymphocyte depletion reverses exacerbated immune-mediated cerebral ischemic brain injury in perioperative stroke mice. Perioperative ischemic stroke triggers aberrant metabolic alterations in peripheral CD8^+^ T cells, in which S-2HG is more abundant. S-2HG alters CD8^+^ T lymphocyte proliferation and differentiation ex vivo and modulates the immune-mediated ischemic brain injury and post-stroke cognitive dysfunction by enhancing CD8^+^ T lymphocyte-mediated neurotoxicity.

**Conclusion:**

Our study establishes that S-2HG signaling-mediated activation and neurotoxicity of CD8^+^ T lymphocytes might exacerbate perioperative ischemic brain injury and may represent a promising immunotherapy target in perioperative ischemic stroke.

**Supplementary Information:**

The online version contains supplementary material available at 10.1186/s12974-022-02537-4.

## Introduction

Perioperative ischemic stroke is one of the most catastrophic complications of surgery, which has strong public health implications [1]. With difficulties in prompt diagnosis, a narrow therapeutic time window, malignant brain edema, and high risks of lethal hemorrhagic transformation, less than 5% perioperative ischemic stroke patients benefit from tPA-mediated thrombolysis [2]. Post-stroke cognitive dysfunction is a common consequence of stroke, resulting in reduced quality of life. Despite medical and technological advances, the incidence of perioperative ischemic stroke and post-stroke cognitive dysfunction has not yet decreased. Therefore, an in-depth investigation of the molecular mechanism involved in perioperative ischemic stroke may be particularly important to the identification of diagnostic and therapeutic targets.

Disruption of immune homeostasis plays a key role in exacerbation of cerebral ischemic stroke. Previous studies have mainly focused on neutrophils, and monocytes in ischemic brain injury [3]. However, the adaptive immune cells-cytotoxic CD8^+^ T lymphocytes are gaining increasing attention in recent years [4]. In response to antigen stimulation or hypoxia, quiescent CD8^+^ T lymphocytes convert to multiple T cell subsets including effector and memory cells [5], but in vivo activation and differentiation in perioperative ischemic stroke remain largely elusive. Moreover, although deleterious effects of proinflammatory cytokines of CD8^+^ T lymphocytes are well characterized, direct neurotoxic effects of brain-infiltrating CD8^+^ T lymphocytes in perioperative ischemic stroke are essentially unknown [6].

The essential roles of immunometabolism in modulating cell fate have gradually begun to be unraveled [7, 8], but context-dependent metabolic effects in vivo remain unclear. Cellular metabolism alters the proliferative effector state of CD8^+^ T lymphocytes [9]. A recent study indicates that hypoxia and mitochondrial deficits may result in S-2HG accumulation, which can regulate the differentiation of CD8^+^ T lymphocytes via altering histone and DNA demethylation and thus trigger adaptive immune responses [10]. Given perioperative risk factors such as surgical insults, traumatic injuries, anesthesia, or hypoxia, there is an urgent need to explore whether and how S-2HG modulates CD8^+^ T lymphocytes and elicit secondary brain damage in perioperative ischemic stroke.

In the present study, we demonstrated that the perioperative stroke mice exhibited exacerbated cerebral ischemic injury and neurological dysfunction more than the stroke-only mice. CD8^+^ T lymphocyte invasion of brain parenchyma and neurotoxicity augment cerebral ischemic injury in the perioperative stroke mice. Perioperative ischemic stroke triggered aberrant metabolic alteration of S-2HG in peripheral CD8^+^ T cells. S-2HG-mediated activation and neurotoxicity of CD8^+^ T lymphocytes might present a novel mechanism and therapeutic target for perioperative ischemic stroke.

## Materials and methods

### Experimental animals

Male C57BL/6 WT mice were obtained from the Chinese PLA General Hospital Laboratory Animal Center and male Rag1^−/−^ mice (B6/JGpt-Rag1^em1Cd^/Gpt) were purchased from GemPharmatech Co., Ltd. All mice used in the study were bred and housed in specific pathogen-free conditions. All animal experiments were undertaken in accordance with the National Institute of Health Guide for Care and Use of Laboratory Animals, with the approval of the Ethics Committee for Animal Experimentation of the Chinese PLA General Hospital. All animals were used at 6–8 weeks of age and randomly assigned for all experiments.

### Mouse model of perioperative ischemic stroke

Ileocecal resection (ICR) and tMCAO surgery were performed to establish the perioperative ischemic stroke model. Animals were anesthetized with inhalational anesthesia using sevoflurane delivered through a nose cone mask. During ICR surgery, ileocecal artery was exposed and ligated with 5-0 silk suture. Identify and divide the ischemic portions of ileum and colon ensuring that blood supply to the transected ends is adequate. Ileum and colon were anastomosed for digestive tract reconstruction with polypropylene 8-0 interrupted sutures. A typical anastomosis will require 14 to 16 interrupted sutures. One day after ICR, perioperative ischemic stroke was induced by transient intraluminal occlusion of the left middle cerebral artery (MCA) with silicone-coated suture (Doccol Corporation) for 60 min followed by reperfusion as described previously [11].

### 7T rodent magnetic resonance imaging scanning

7T/400 mm ultra-high field magnetic resonance system (BioSpec70/20USR, Bruker Corporation) was performed to evaluate the cerebral infarct volume in live mice. Mice were anesthetized with 2.5–3.5% isoflurane, while vitals signs were continually monitored with the monitoring system during the examinations. T2-weighted images were captured using relaxation enhancement sequences. Imaging parameters were as follows: repetition time (TR) = 3500 ms, effective echo time (TE) = 40 ms, slice thickness = 0.5 mm, field of view (FOV) = 15 × 15 mm, matrix size = 120 × 120. Infarct volumes were calculated as scanned volumes of contralateral brain tissue minus scanned volumes of the non-infarcted areas of the ipsilateral lesioned brain tissue using Image J software (NIH).

### Untargeted metabolomic profiling of CD8^+^ T lymphocytes by LC–MS/MS

Isolate untouched and highly purified CD8^+^ T cells from stroke mouse splenocytes by using EasyStep Mouse CD8^+^ T Cell Isolation Kit (StemCell Technologies). The analysis process of untargeted metabolomics was divided into two parts: experimental and bioinformatics analysis. The cell metabolites were extracted using the methanol:acetonitrile:water extraction protocol and then analyzed by liquid chromatography system coupled with high-resolution mass spectrometer (Thermo Fisher Scientific, USA). The bioinformatics analysis mainly included: data preprocessing, data quality control, statistical analysis, screening for differential metabolites, and pathway enrichment analysis.

### Lentiviral transfection of CD8^+^ T lymphocytes

L-2-Hydroxyglutarate dehydrogenase (L2hgdh) overexpression was achieved by transfecting lentiviral particles expressing a Flag epitope-tagged form of murine L2hgdh (pSLenti-EF1-EGFP-CMV-L2hgdh-3xFLAG-WPRE, denoted as L2hgdh-Flag) into CD8^+^ T cells 72 h before experiments. pSLenti-EF1-EGFP-CMV-CHRNA7(GV417)-3FLAG (denoted as empty vector) was used as control lentivirus. L2hgdh knockdown was generated by transfecting lentivirus expressing L2hgdh-specific short hairpin RNAs (denoted as shL2hgdh) into CD8^+^ T cells 72 h before experiments. Lentiviral vector expressing a scrambled sequence (shScramble) was used as control shRNA. The lentiviral vectors containing L2hgdh-Flag, empty vector, shL2hgdh, and shScramble were purchased from Obio (Shanghai, China).

### Adoptive cell transfer of Rag1^−/−^ mice

CD8^+^ T lymphocytes isolated from healthy mice spleen using magnetic sorting (StemCell Technologies) were plated at 5 × 10^5^ per well in a 24-well plate with anti-CD3/CD28 bead-based stimulation (Miltenyi) for 48 h. After being transfected with lentivirus L2hgdh-Flag to upregulate L2hgdh expression, 2 × 10^6^ CD8^+^ T cells were injected via femoral vein to recipient Rag1^−/−^ mice before the perioperative stroke model established.

### Behavioral tests

All behavioral tests were carried out by the blinded experimenter. The modified Garcia score test [12, 13] and foot fault test [14] were performed as described previously to assess sensorimotor deficits. Morris water maze (MWM) was used to investigate spatial learning, reference memory, and working memory. The three-chamber paradigm test for sociability and social novelty preference was performed to assess sociability.

### Antibodies for western blot and immunofluorescent staining

The primary antibodies used for western blot are as follows: rabbit anti-HIF-1α (1:1000, Cell Signaling Technology, RRID: AB_2799095), and rabbit anti-LDHA (1:5000, Abcam, RRID: AB_2889291). For western blotting, horseradish peroxidase (HRP)-conjugated secondary antibodies (1:10,000, Sigma-Aldrich) were used. The primary antibodies used for immunofluorescent staining are as follows: rabbit anti-CD8α (1:200, Abcam, RRID: AB_2890649), rabbit anti-CD4 (1:100, Abcam, RRID: AB_2686917), rabbit anti-GFAP (1:200, Cell Signaling Technology, RRID: AB_2799963), and rabbit anti-Iba1 (1:200, Cell Signaling Technology, RRID: AB_2820254). The secondary antibodies used for immunohistochemical staining were bought from Invitrogen.

### Antibodies for flow cytometry analysis

Antibodies used to perform flow cytometry staining were all purchased from BioLegend unless otherwise noted. The antibodies are as follows: PerCP/Cy 5.5-anti-mouse CD45 (30-F11, RRID: AB_893344), FITC anti-mouse CD8a (QA17A07, RRID: AB_2750210), PE anti-mouse CD44 (IM7, RRID:AB_493686), APC-anti-mouse CD62L (MEL-14, RRID: AB_313098), PerCP/Cy 5.5-anti-mouse CD8 (53-6.7, RRID: AB_2075239), FITC anti-mouse CD4 (GK1.5, RRID: AB_312690), APC anti-mouse Ly-6G/Ly-6C (Gr-1) (RB6-8C5, RRID: AB_313376), PE anti-mouse CD45R/B220 (RA3-6B2, RRID: AB_312992). FlowJo V10 (Tree star) were performed to analyze flow cytometry data acquired from BD FACSDiva flow cytometry.

### Assessment of infarct volume

To evaluate the extent of infarct area, brains were sectioned and incubated with 2.0% 2,3,5-triphenyltetrazolium chloride staining (TTC, Sigma-Aldrich) as previously described [15]. Microtubule-associated protein 2 (MAP2) staining was used to delineate infarct area with rabbit anti-MAP2 antibodies (1:200, Cell Signaling Technology). The stained sections were scanned, and infarct areas were measured using Image J software (NIH). Relative infarct volumes with correction for cerebral edema were assessed based on the following equation: (volumes of contralateral brain tissue minus volumes of the non-infarcted areas of the ipsilateral lesioned brain tissue).

### Evans blue administration and quantification

Mice were injected intravenously with 4 ml/kg of 2% Evans Blue dye (Sigma-Aldrich), followed by a 3-h circulation in vivo before euthanasia. The brain tissue was minced and sonicated in *N*,*N*-dimethylformamide (10 ml/kg, Sigma-Aldrich), incubated for 24 h at 60 °C, and centrifuged at 3000 rpm for 10 min [16]. The supernatants were obtained and analyzed at 620 nm using spectrophotometer (DU-640 spectrophotometer, Beckman Coulter).

### RT-PCR and quantitative PCR analysis

Total RNA of occludin was extracted and purified using TRIzol reagent (Invitrogen). 1 μg of total RNA was reversed transcribed into complementary DNA using the Superscript III system (Invitrogen). Quantitative real-time PCR was carried out with 7500 Real Time PCR system (Applied Biosystems), and the relative expression was calculated using the 2^ΔΔCt^ method.

### HE staining, Nissl staining, Tunel staining and NeuN staining

HE staining, Nissl staining, Tunel staining and NeuN staining were performed to assess the ischemia-induced neuronal damage in the penumbra 7 days after stroke. The brain tissue was embedded in paraffin and coronally sectioned to a thickness of 4 μm. HE^+^, Nissl^+^, Tunel^+^ or NeuN^+^ cells were counted in 5 different areas of the ischemic penumbra by the blinded investigators.

### CD8^+^ T cell proliferation assay

CD8^+^ T lymphocytes purified from healthy mice spleen by using EasyStep Mouse CD8^+^ T Cell Isolation Kit (StemCell Technologies) were cultured with anti-CD3/CD28 antibodies and IL-2 and then treated with S-2HG, lentivirus L2ghdh-Flag, or lentivirus shL2hgdh. After incubation, Edu (5-ethynyl-2′-deoxyuridine) (Cell-Light Edu Apollo567 In Vitro Kit, RiboBio) was added and stained for 2 h. Confocal imaging (FV1000, Olympus, Japan) were performed to assess Edu labeling with proliferating CD8^+^ T cells.

### Statistical analyses

All results were analyzed by an investigator who was blinded to the study protocol. All values are presented as mean ± standard error of the mean (SEM) for continuous numerical variables. Experimental group sizes were predetermined based on previous similar studies (power 0.8, α 0.05). Student’s t-test was performed to compare the difference between two groups. When comparing multiple groups, one-way ANOVA was applied in the statistical analysis, with post hoc Bonferroni correction for multiple comparisons. Comparisons of different groups in each time point were carried out using two-way ANOVA plus post hoc Bonferroni test. We performed statistical analyses using GraphPad Prism 9 (GraphPad Software). All statistical tests were two-tailed and *P* values < 0.05 were deemed statistically significant.

## Results

### The perioperative stroke mice exhibit more severe ischemia-induced cerebral ischemic injury than the stroke-only mice

**Fig. 1:**
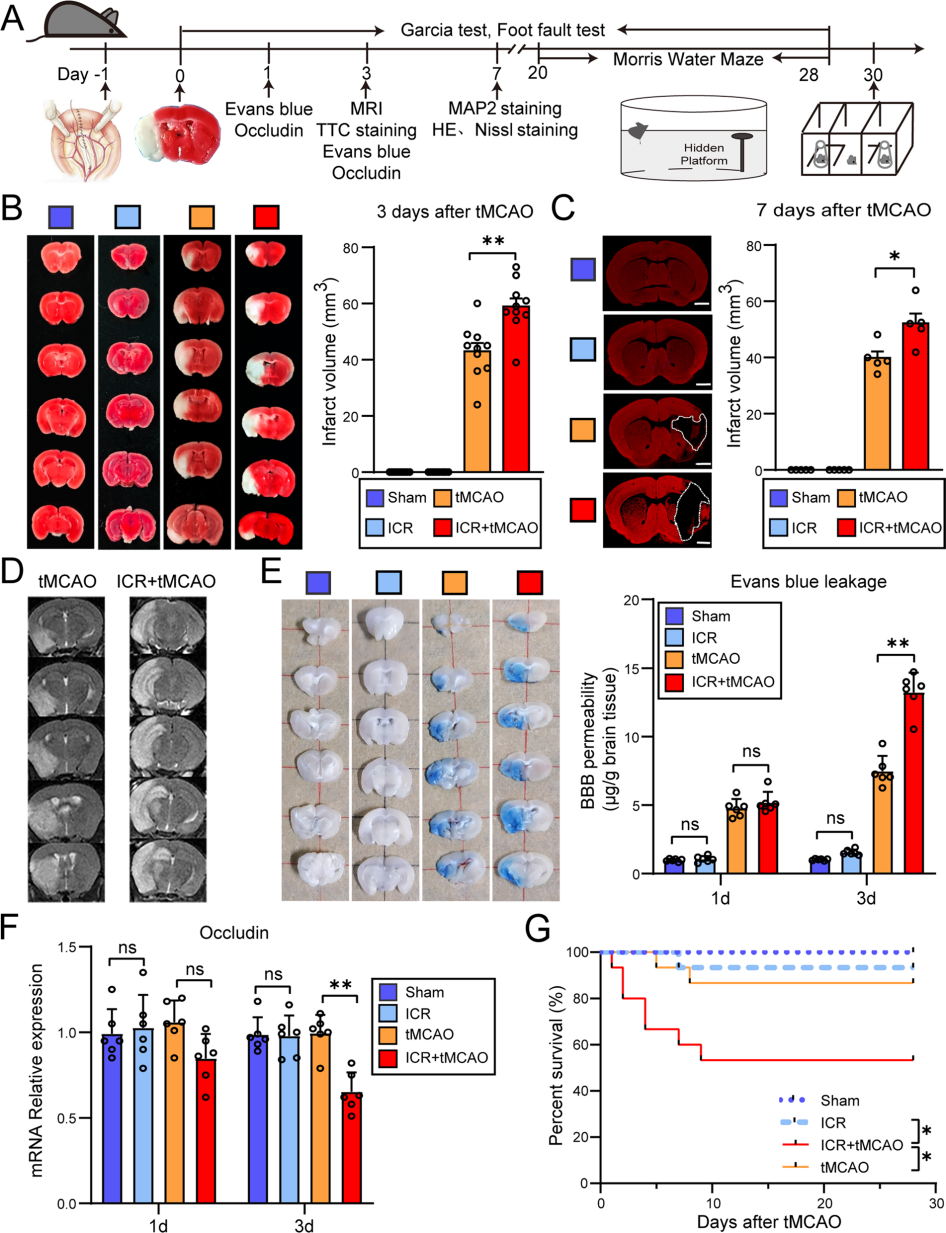
The perioperative stroke mice exhibit more severe cerebral ischemic injury than the stroke-only mice. **A** Flowchart illustrates the experimental design. **B** Representative images and quantification of TTC-stained coronal sections showing the infarct volume 3 days after stroke (*n* = 10/group). **C** Representative images and quantification of MAP2-stained coronal slices showing the infarct volume 7 days after stroke (*n* = 5/group). Scale bars = 1 mm. **D** Representative T2-weighted images (T2WI) of mice brain showing ischemic lesion. **E** Representative images and quantification of Evans blue extravasation (1 and 3 days after stroke) (*n* = 6/group). **F** Quantification of mRNA expression of tight junction protein occludin (1 and 3 days after stroke) (*n* = 6/group). **G** Kaplan–Meier estimates of survival until 28 days after tMCAO (*n* = 15/group). The sham mice were defined as the mice that underwent laparotomy without ICR. *P < 0.05, **P < 0.01, ns indicates nonsignificant

To investigate how perioperative ischemic stroke influences cerebral injury, we established the perioperative stroke model and quantified the brain injury on infarct size by TTC staining and MAP2 staining (Fig. 1A). Compared with stroke-only mice (tMCAO group), the perioperative stroke mice (ICR + tMCAO group) exhibited significantly increased infarct volume by TTC staining (*t*_(18)_ = 3.789, *P* = 0.0013, Fig. 1B) (3 days following ischemia) and by MAP2 staining (*t*_(8)_ = 2.925, *P* = 0.0191, Fig. 1C) (7 days following ischemia). Meanwhile, we observed that the edema and infarct territories were highlighted using a T2-weighted imaging sequence of 7T rodent magnetic resonance imaging scanning (Fig. 1D). Compared with stroke-only mice, the proportion of HE^+^ (*P* < 0.0001), Nissl^+^ (*P* < 0.0001) and NeuN^+^ (*P* = 0.0097) cells in the penumbra of ischemic hemisphere were significantly diminished in mice with perioperative stroke. Meanwhile, perioperative stroke mice showed an obvious augment of Tunel^+^ cells (*P* < 0.0001) in the ischemic penumbra, suggesting a worsen neuronal injury (Additional file 1: Fig. S1). We performed the blood–brain barrier (BBB) permeability experiment using Evans blue administration intravenously in vivo. We confirmed that the perioperative stroke mice exhibited an augment of BBB breakdown at 3 days following ischemia, as indicated by the quantification of Evans blue leakage (*P* < 0.0001, Fig. 1E). Next, as occludin is a critical molecule for preserving the BBB permeability, we demonstrated that the perioperative stroke mice exhibited more severe occludin loss in the ischemic penumbra when assayed at 3 days following tMCAO (*P* < 0.0001, Fig. 1F). Notably, the perioperative stroke mice demonstrated dramatically augmented mortality within 28 days following ischemia compared to stroke-only mice, as indicated in Kaplan–Meier (KM) survival curves (log-rank *χ*^2^ = 16.10, *P* = 0.0011, Fig. 1G). Taken together, these findings suggest that ischemia-induced cerebral ischemic injury in the perioperative stroke mice is more severe compared to the stroke-only mice.

### The perioperative stroke mice are more vulnerable to sensorimotor, cognitive, and social dysfunction

**Fig. 2:**
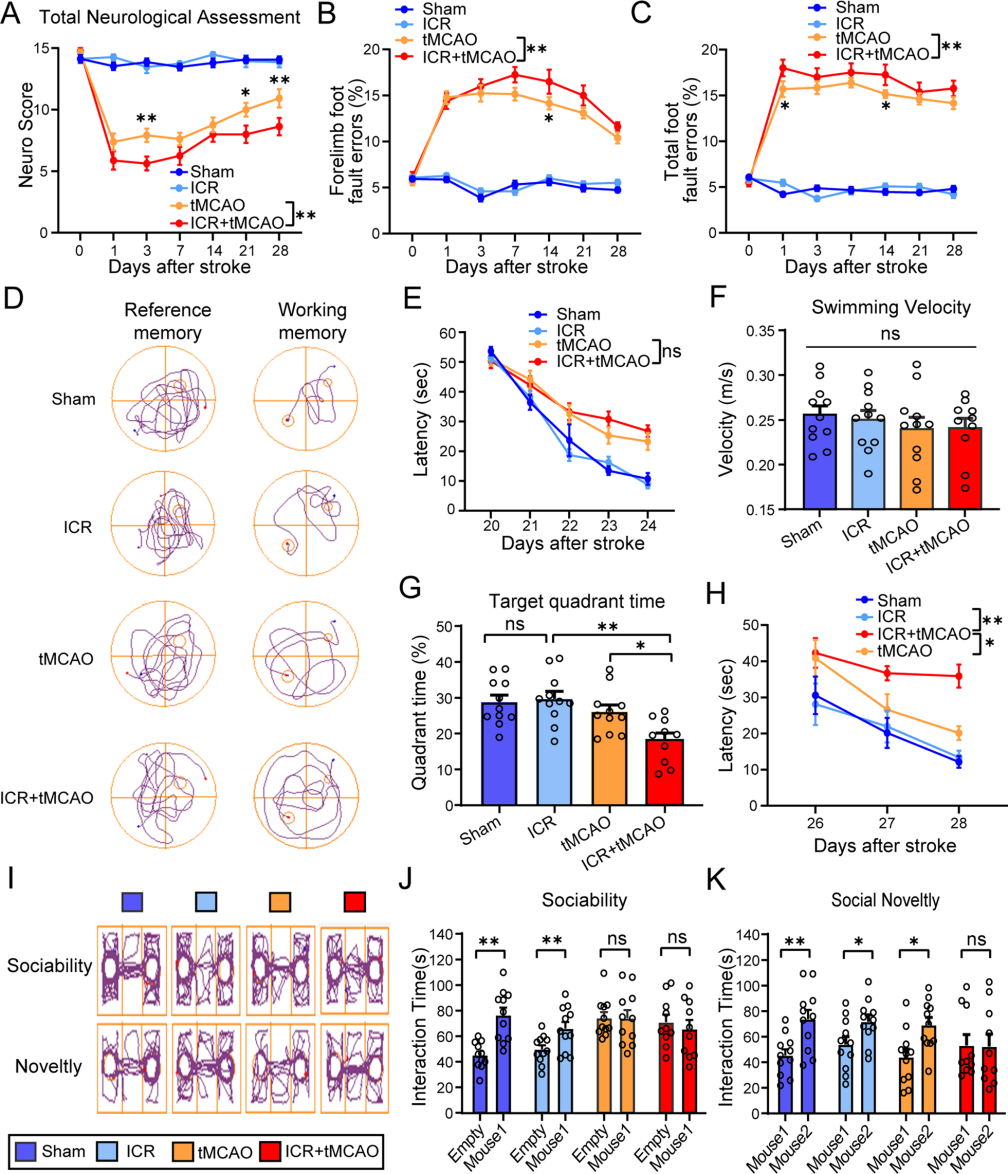
The perioperative stroke mice develop profound neurological deficits such as sensorimotor, cognitive, and social dysfunction. **A**–**C** Sensorimotor function was assessed using Garcia score test (**A**) or foot fault test (**B**, **C**) until 28 days after tMCAO (*n* = 10–15/group). **D** Representative trajectories of mice showing spatial reference memory or spatial working memory. **E** Spatial learning in the navigation test of MWM on days 20, 21, 22, 23, 24 after stroke. Average escape latency is shown for the training sessions. **F** Quantification of swimming speed during probe test on day 25 after stroke. **G** Time traveled in the target quadrant during the probe test (*n* = 10–11/group). **H** Quantification of latency to the platform during spatial working memory testing on days 26, 27, and 28 after stroke (*n* = 10–11/group). **I** Representative trajectories of mice showing sociability and social novelty preference. **J** During the sociability test, the time spent interacting with a stranger mouse or with an empty cup was recorded on days 30 after stroke (*n* = 10–11/group). **K** During the social novelty preference test, the time spent interacting with a novel introduced stranger versus an initial stranger was recorded on days 30 after stroke (*n* = 10–11/group). *P < 0.05, **P < 0.01, ns indicates nonsignificant

We next performed the modified Garcia score test and foot fault test to assess sensorimotor deficits 0, 1, 3, 7, 14, 21, and 28 days after ischemia onset, respectively. Sensorimotor impairments were robustly exacerbated in perioperative stroke mice compared with stroke-only mice, as proved by neurobehavioral changes on the modified Garcia score test for total neurological assessments (*P* < 0.0001) including gross motor, body proprioception, and motor coordination (Fig. 2A and Additional file 1: Fig. S2). Moreover, we observed an apparent difference in both forelimb foot fault errors (*P* = 0.0006) and total foot fault errors (*P* = 0.0003) between perioperative stroke mice and stroke-only mice, indicating a more severe fine motor impairment in perioperative stroke mice (Fig. 2B, C).

We then used Morris water maze to investigate whether perioperative ischemic stroke would affect spatial learning, reference memory, and working memory. In both perioperative stroke mice and stroke-only mice, the escape latency prolonged significantly compared to sham mice from day 20 to day 24 post-ischemia, suggesting that the spatial learning ability declined after stroke. However, perioperative stroke mice presented with only an increasing trend with respect to spatial learning impairment without statistical significance as compared with stroke-only mice (*P* = 0.8251, Fig. 2E). On day 25 poststroke, no significant difference was detected in swimming velocity among groups in the probe test (*F*_(3, 39)_ = 0.4777, *P* = 0.6997), suggesting that the performance on MWM test was not compromised by differences in locomotor deficits (Fig. 2F). Interestingly, the mice with perioperative stroke had significant deficits in the ability to remember the exact platform location during the probe test and spent less time searching in the target quadrant than stroke-only mice (*F*_(3, 39)_ = 6.727, *P* = 0.0009), indicating a worse spatial reference memory (Fig. 2D, G). On days 26 to 28 post-ischemia, we performed spatial working memory test by randomly changing the hidden platform and initial entry quadrant of the mice tested. More severe impairment in spatial working memory in perioperative stroke mice was verified (*P* = 0.0362), as indicated by a relatively higher escape latency compared with stroke-only mice (Fig. 2D, H).

Since previous animal and human studies had reported that the stroke or a spectrum of neurodegenerative diseases could lead to high risks of social dysfunction [17], we hypothesized that perioperative ischemic stroke could affect social interaction behaviors. The three-chamber paradigm test for sociability and social novelty preference has been successfully employed to assess sociability in mice [18, 19]. In sociability test, the perioperative stroke mice exhibited no preference for stranger mice (mouse 1) and empty cup at 30 days following ischemia, indicating impaired sociability (*t*_(18)_ = 0.1570, *P* = 0.8770, Fig. 2I, J). In social novelty preference test, the perioperative stroke mice showed no preference for a novel encountered stranger (mouse 2) and an initial stranger (mouse 1) at 30 days poststroke, indicating impaired social novelty preference (*t*_(18)_ = 0.6937, *P* = 0.4967, Fig. 2I, K). These findings indicate that perioperative stroke mice contributed to sensorimotor, cognitive, and social dysfunction, suggesting that related brain regions or structural and functional connectivity between brain areas may be disrupted due to the larger range of infarct volume.

### CD8^+^ T lymphocyte invasion of brain parenchyma and direct neurotoxicity augment in the perioperative stroke mice

**Fig. 3:**
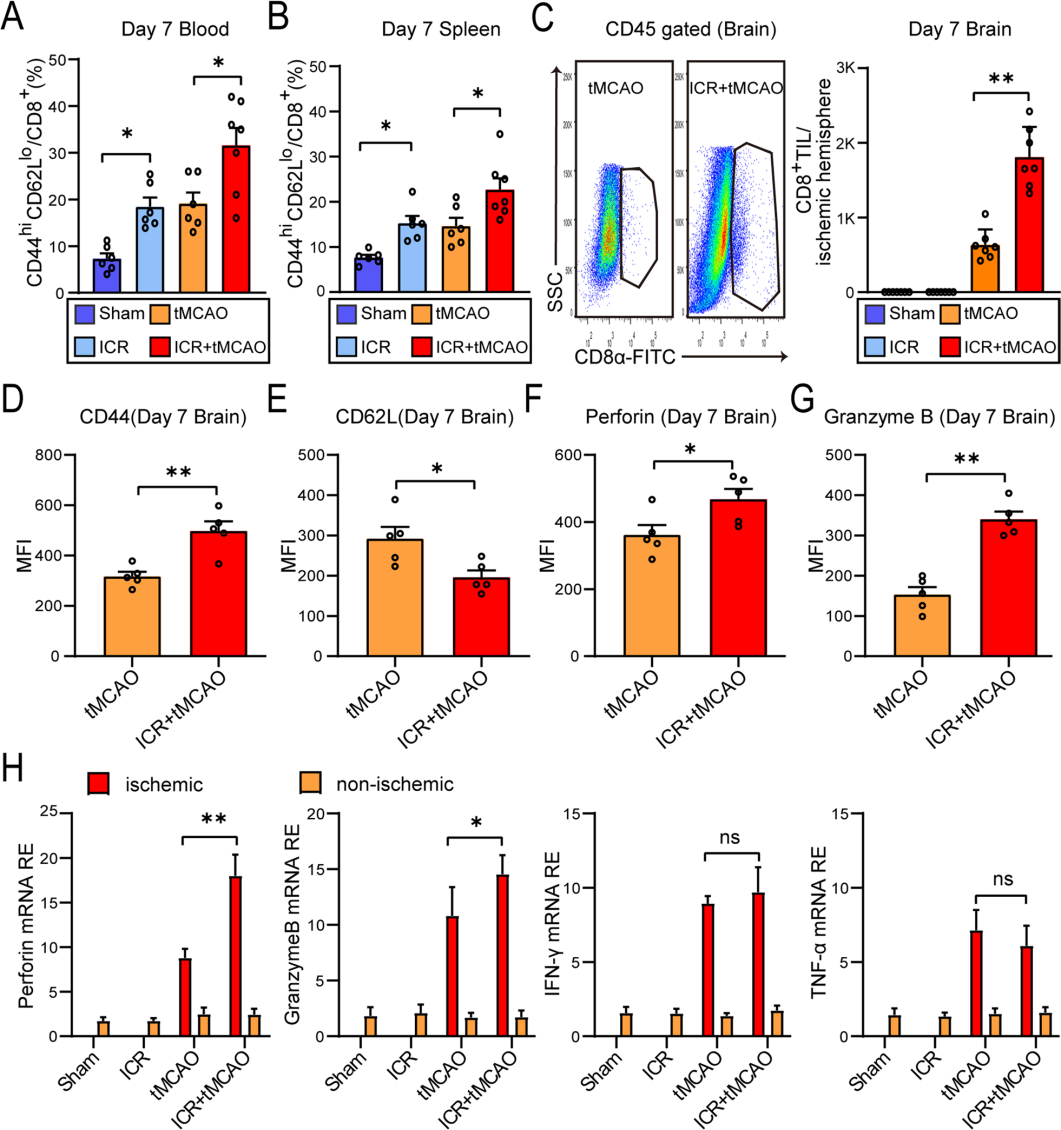
The activation and brain invasion of CD8^+^ T lymphocytes exacerbate ischemic brain injury in perioperative stroke mice. **A**, **B** Flow cytometry analysis on CD44^hi^CD62L^lo^ percentage on CD8^+^ T lymphocytes in blood (**A**) and spleen (**B**) 7 days after stroke (*n* = 6–7/group). **C** Representative dot plots and an absolute number of brain-invading CD8^+^ T cells 7 days after stroke (*n* = 7/group). **D**–**G** CD44 (**D**), CD62L (**E**), Perforin (**F**), and Granzyme B (**G**) mean fluorescence intensity (MFI, arbitrary unit) of brain infiltrating CD8^+^ T cells 7 days after stroke (*n* = 5/group). **H** mRNA levels of cytokines Perforin, Granzyme B, IFN-γ, and TNF-α as measured by RT-PCR in ischemic and non-ischemic hemispheres (*n* = 5/group). The sham mice were defined as the mice that underwent laparotomy without ICR. *P < 0.05, **P < 0.01, ns indicates nonsignificant

Immune cells are recognized as key players in exacerbation of ischemic brain injury. Considering that profound immune responses can be rapidly activated during the perioperative period or ischemic stroke, we next clarified which cell subsets conducted the exacerbation effect of cerebral ischemic injury in perioperative stroke mice and focused on the direct cytotoxic effects of the brain-infiltrating immune cells. To this end, we assessed the major subset of immune cells at 7 days after stroke by flow cytometry (FCM) analysis. Compared to Sham mice, ICR mice showed an obvious augment of CD44^hi^CD62L^lo^CD8^+^ T lymphocytes in blood (*P* = 0.0365) and spleen (*P* = 0.0401), demonstrating an active functional status of peripherally CD8^+^ T lymphocytes. Compared to stroke-only mice, the percentage of activated CD8^+^ T lymphocytes in blood (*P* = 0.0131) and spleen (*P* = 0.0223) increased significantly in perioperative stroke mice, indicating a peripherally increasing activation state of CD8^+^ T lymphocytes in perioperative stroke model (Fig. 3A, B). Notably, greatly increased brain-invading CD8^+^ T lymphocytes (*F*_(3,24)_ = 6.398, *P* < 0.0001) were detected in perioperative stroke mice by the flow analysis of ischemic hemisphere, suggesting that the perioperative stroke led to CD8^+^ T lymphocytes recruitment to brain parenchyma, which could potentially enhance the neurotoxic effect (Fig. 3C). Meanwhile, perioperative stroke mice showed an obvious augment of CD44^hi^CD62L^lo^CD8^+^ T lymphocytes in the ischemic hemisphere, demonstrating an active functional status of brain-infiltrating CD8^+^ T lymphocytes (Fig. 3D, E). Moreover, brain-infiltrating CD8^+^ T lymphocytes of perioperative stroke mice responded with more Perforin (*t*_(8)_ = 2.481, *P* = 0.0380) and Granzyme B (*t*_(8)_ = 7.065, *P* < 0.0001), which can exert cytotoxicity to neurons [20] (Fig. 3F, G). Cerebral mRNA expressions of Perforin (*t*_(8)_ = 3.553, *P* = 0.0075) and Granzyme B (*t*_(8)_ = 2.680, *P* = 0.0279) in perioperative stroke mice were obviously higher than stroke-only mice 7 days after stroke onset, whereas the mRNA level of IFN-γ (*t*_(8)_ = 0.9686, *P* = 0.3611) and TNF-α (*t*_(8)_ = 1.208, *P* = 0.2617) did not reflect abnormal variability (Fig. 3H). These suggested the direct neurotoxicity of CD8^+^ T lymphocyte plays a more important role in perioperative ischemic brain injury than humoral pathways (IFN-γ or TNF-α). In contrast, the flow cytometry analysis of brain-infiltrating CD4^+^ T lymphocytes (*t*_(9)_ = 0.6418, *P* = 0.5370), B cell (*t*_(9)_ = 1.332, *P* = 0.2157) and neutrophil (*t*_(9)_ = 1.394, *P* = 0.1969) did not reveal a significant difference between perioperative stroke mice and stroke-only mice, respectively (Additional file 1: Fig. S3A–F). In addition, we further performed immunofluorescence staining to evaluate the invasion of CD8^+^ T lymphocytes or CD4^+^ T lymphocytes in the ischemic brain 7 days after stroke onset. The enrichment of brain-invading CD8^+^ T cells (*F*_(3, 16)_ = 61.43, *P* < 0.0001, vs. tMCAO group) in the ischemic hemisphere was more evident in perioperative stroke mice (Additional file 1: Fig. S4A, B). However, the perioperative stroke mice did not show an obvious enhancement in recruiting CD4^+^ T lymphocytes (*F*_(3, 16)_ = 15.43, *P* = 0.9973, vs. tMCAO group) to the ischemic hemisphere (Additional file 1: Fig. S4A, C). We then explored the activation effects of astrocytes and microglia in ischemic penumbra, respectively. Differences between perioperative stroke and stroke-only mice in astrocytic (*P* = 0.0697) and microglial (*P* = 0.0684) activation were statistically insignificant (Additional file 1: Fig. S4A, D). Collectively, these results suggest that the invasion of brain parenchyma and direct neurotoxicity of CD8^+^ T lymphocytes may play a critical role in immune-mediated cerebral ischemic injury and contribute to the exacerbation of ischemic brain injury in perioperative stroke mice.

### Neuroprotection of CD8^+^ T lymphocyte depletion in perioperative stroke mice

**Fig. 4:**
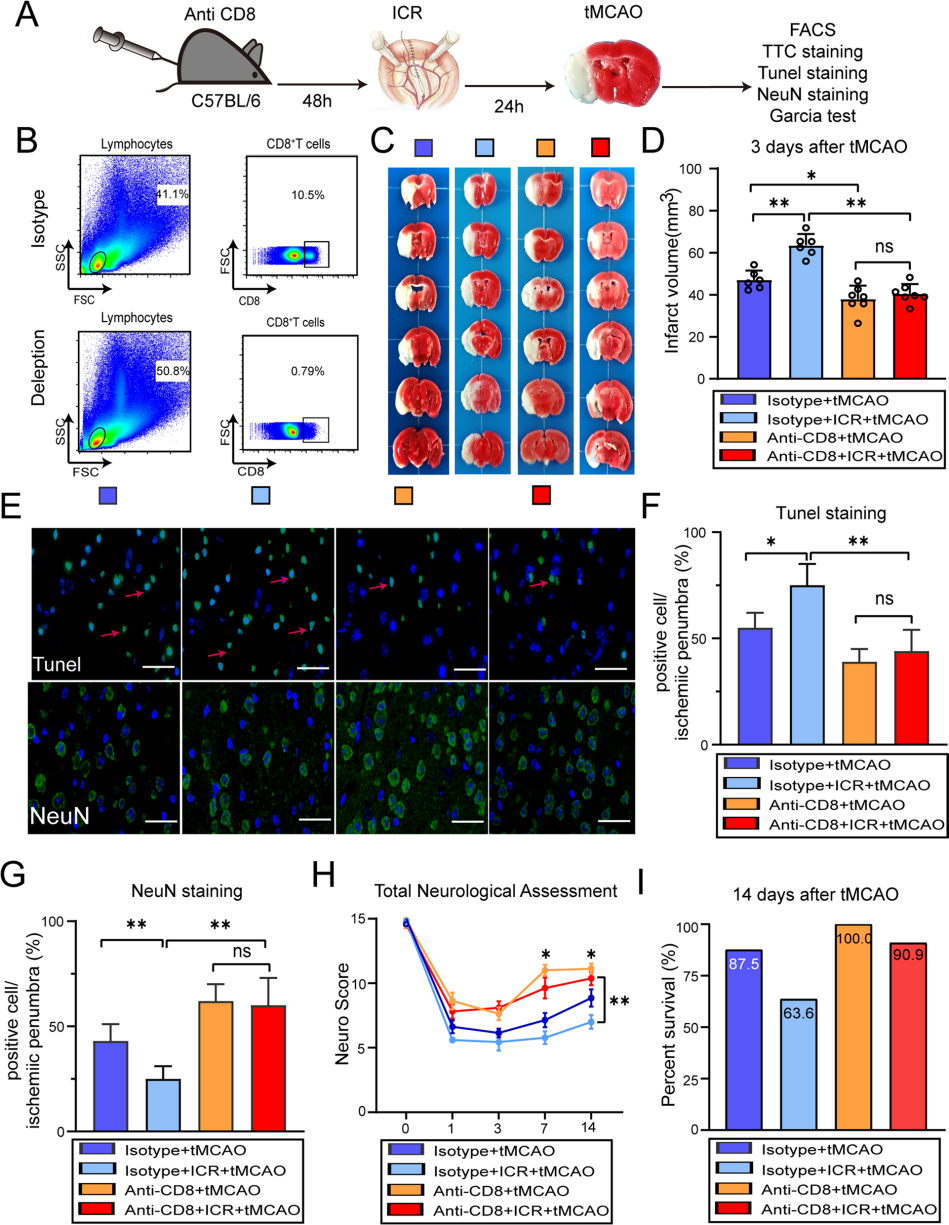
CD8^+^ T lymphocytes depletion is crucial for the reduction of ischemic brain injury in perioperative stroke mice. **A** Flowchart illustrates the experimental design. Three days before tMCAO, C57BL/6 mice were injected intraperitoneally with 100 μg InVivoMAb anti-mouse CD8α antibody or 100 μg Rat IgG2b (isotype antibody). **B** Representative dot plots of flow cytometry of CD8^+^ T cells in the spleen after CD8^+^ T lymphocytes depletion. **C**, **D** Representative and quantification of TTC staining in 6 consecutive coronal sections (1 mm apart) 3 days after stroke (*n* = 6–7/group). **E** Representative images of brain tissue with Tunel staining or NeuN staining in the ischemic penumbra 7 days after stroke. Red arrows signify the injured neurons. Scale bar = 20 μm. **F**, **G** Quantification of Tunel^+^ cells (**F**) or NeuN^+^ cells (**G**) (*n* = 5/group). **H** Neurological deficits were evaluated until 14 days after tMCAO (*n* = 8–11/group). **I** The survival rate of subject mice after CD8^+^ T lymphocytes depletion during 14 days after tMCAO (*n* = 8–11/group). *P < 0.05, **P < 0.01, ns indicates nonsignificant

Having determined that the active status of CD8^+^ T lymphocytes increased both peripherally and centrally in perioperative stroke mice, we next sought to elucidate the direct effect of CD8^+^ T lymphocytes on brain infarct size and neurobehavioral deficits after tMCAO in perioperative stroke mice by in vivo depletion of CD8^+^ T cell populations using anti-CD8 monoclonal antibodies (mAb) (Fig. 4A). Flow cytometry analysis confirmed that splenic CD8^+^ T lymphocytes were depleted in anti-CD8mAb-treated mice, whereas splenic CD8^+^ T cell populations were not diminished by the isotype IgG treatment (Fig. 4B). Three days after stroke, administration of anti-CD8α mAb led to reduced brain infarct size in perioperative stroke mice (*P* < 0.0001) and stroke-only mice (*P* = 0.0262) compared with isotype IgG-treated mice (Fig. 4C, D). Interestingly, we further compared brain infarct size between perioperative stroke mice with anti-CD8α mAb and stroke-only mice with anti-CD8α mAb and observed no measurable differences between these two groups (*P* = 0.8075), indicating a therapeutic potential for exacerbated cerebral ischemic brain damage (Fig. 4D). Moreover, compared with isotype IgG-treated mice, administration of anti-CD8α mAb lead to reduced Tunel^+^ cells (*P* < 0.0001) and increased NeuN^+^ cells (*P* < 0.0001) in the ischemic penumbra with perioperative stroke mice (Fig. 4E–G). By 7 days and 14 days after ischemia, neurobehavioral dysfunctions were significantly ameliorated in perioperative stroke mice by depletion of CD8^+^ T lymphocytes as compared to isotype IgG-treated mice (*P* < 0.0001, Fig. 4H). Meanwhile, there was a trend toward improved survival for anti-CD8α-treated mice (Fig. 4I). Taken together, these results demonstrate that CD8^+^ T lymphocyte depletion reverses exacerbated immune-mediated cerebral ischemic brain injury and is crucial for the reduction of brain infarct volume, protection of neurons and remission of neurobehavioral deficits in perioperative stroke mice.

### Immunometabolite S-2HG upregulation in CD8^+^ T cell after perioperative ischemic stroke

**Fig. 5:**
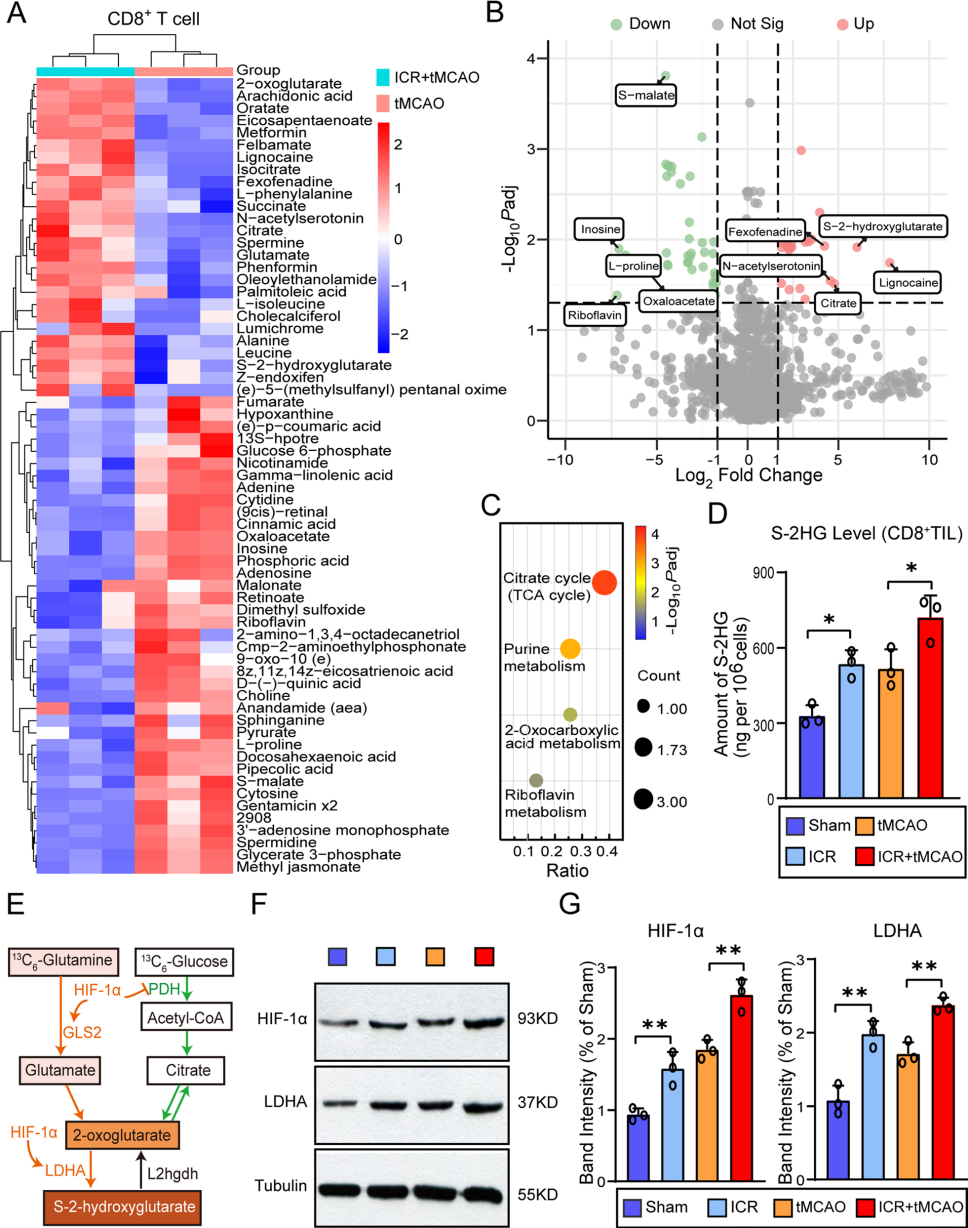
Accumulation of immunometabolite S-2HG in CD8^+^ T lymphocytes after perioperative ischemic stroke. **A** Heat map of all differentially expressed intracellular metabolites. The differential expressed metabolites were confirmed with a fold change distribution of ICR + tMCAO/tMCAO > 2.0 or < 0.5. **B** Volcano plot indicating the metabolites of differential accumulation [log_2_ (fold change) on *X*-axis] and significant change [− log_10_ *P*_adj_ on *Y*-axis] in the ICR + tMCAO and tMCAO group. **C** KEGG analysis of enriched biological processes with differential expressed metabolites. **D** S-2HG levels in splenic CD8^+^ T cells sorted from perioperative stroke mice or stroke-only mice 3 days after tMCAO (*n* = 3/group). **E** Schematic showing the physiological and pathophysiological mechanism of S-2HG production, listing representative enzymes, and metabolic molecules. **F**, **G** Representative western blot images and quantification of HIF-1α or LDHA 3 days after stroke in the sorted splenic CD8^+^ T cells from perioperative stroke mice or stroke-only mice (*n* = 3/group). The sham mice were defined as the mice that underwent laparotomy without ICR. *P < 0.05, **P < 0.01

Metabolic dysregulation has been widely associated with immune cell production and differentiation [21–23]. Unlike non-perioperative stroke, perioperative stroke has a distinct characteristic: surgical intervention [24]. Given that surgical insults, traumatic injuries, or inflammatory stress events exist during the perioperative period, which are important for triggering aberrant metabolic alterations [25], we hypothesized that metabolic responses are essential for CD8^+^ T cells to cope with the demand for cell growth and multiple rounds of division. To test this, we established the perioperative stroke model and performed untargeted metabolomics on sorted splenic CD8^+^ T lymphocytes 3 days after tMCAO. Consistently, partial least-squares discrimination analysis (PLS-DA) (data not shown) and unsupervised hierarchical clustering indicated that the metabolomic profiling of perioperative stroke mice was significantly different compared to stroke-only mice (Fig. 5A). Among a total of 1811 metabolites, 25 upregulated metabolites and 40 downregulated metabolites were detected in perioperative stroke mice. The volcano plot further showed that the top 5 upregulated differential metabolites and the top 5 downregulated differential metabolites represented in the perioperative stroke and stroke-only mice, including lignocaine, S-2-hydroxyglutarate, citrate, *N*-acetylserotonin, fexofenadine, inosine, riboflavin,* S*-malate, oxaloacetate, and l-proline (Fig. 5B). KEGG pathway analysis with hierarchical clustering revealed that the differential metabolites in perioperative stroke mice were mainly enriched in early citrate cycle (TCA cycle) metabolism (Fig. 5C). Notably, S-2HG was more abundant in CD8^+^ T cells of perioperative stroke mice than that in stroke-only mice. Moreover, we confirmed that S-2HG in CD8^+^ T cells significantly increased in ICR mice (*P* = 0.0233, vs. Sham group) and perioperative stroke mice (*P* = 0.0263, vs. tMCAO group) by quantitative liquid chromatography–mass spectrometry (LC–MS), indicating a strong association between the accumulation of S-2HG and the activation of CD8^+^ T cells (Fig. 5D). Previous studies [9] suggest that hypoxia-inducible factor 1α (HIF-1α) protein accumulates in the context of hypoxia or stress, and induces the overexpression of lactate dehydrogenase A (LDHA), and elevates the production of S-2HG (Fig. 5E). By 3 days after tMCAO, we next elucidated the expression of HIF-1α and LDHA in isolated CD8^+^ T lymphocytes using Western blot. HIF-1α (*F*_(3, 8)_ = 45.59, *P* < 0.0001) and LDHA (*F*_(3, 8)_ = 33.37, *P* < 0.0001), known to be important for S-2HG production and accumulation, strikingly increased in perioperative stroke mice compared with stroke-only mice (Fig. 5F, G). Collectively, we conclude that CD8^+^ T cells undergo profound metabolic alterations in perioperative stroke mice, with an aberrant accumulation of immunometabolite S-2HG.

### S-2HG alters CD8^+^ T lymphocyte proliferation and differentiation

**Fig. 6:**
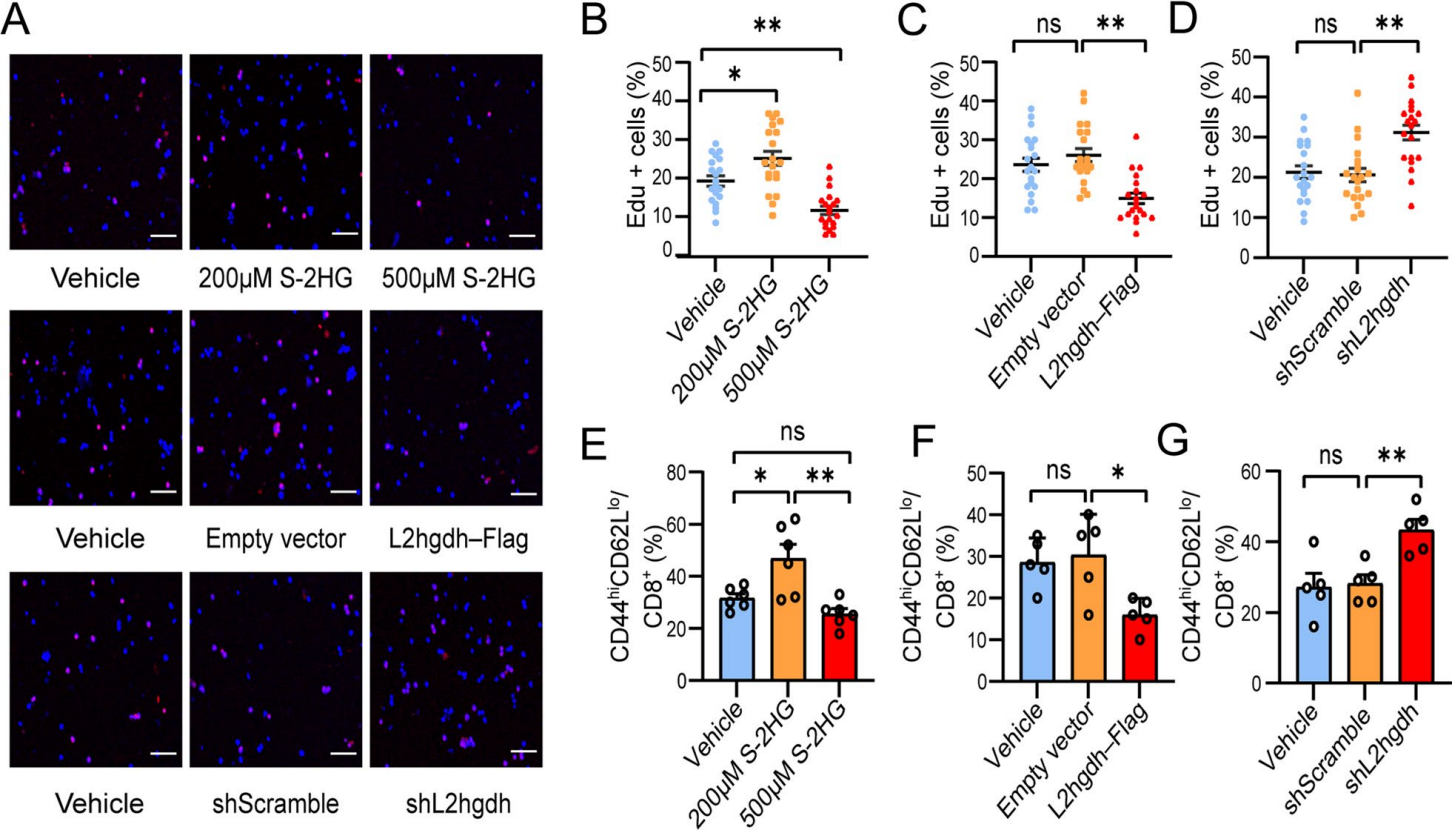
S-2HG alters CD8^+^ T cell proliferation and activation. **A** Representative immunostaining of CD8^+^ T cells treated with vehicle, 200 μM S-2HG, 500 μM S-2HG, empty vector, L2ghdh-Flag, shScramble, shL2hgdh followed by Edu pulse labeling. Scale bar = 10 μm. **B**–**D** Percentage of Edu-positive cells in CD8^+^ T cells treated with S-2HG (**B**), L2ghdh-Flag (**C**), or shL2hgdh (**D**). **E**–**G** Flow cytometry analysis on CD44^hi^CD62L^lo^ percentage on CD8^+^ T cells treated with S-2HG (**E**), L2ghdh-Flag (**F**), and shL2hgdh (**G**). *P < 0.05, **P < 0.01, ns indicates nonsignificant

Next, we made efforts to test whether and how S-2HG directly alters CD8^+^ T lymphocytes proliferation and differentiation. Homeostatic proliferation and differentiation were evaluated by Edu incorporation assay and flow cytometric analysis, respectively (Fig. 6A). We first separated CD8^+^ T lymphocytes from healthy C57BL/6 mice spleen using magnetic sorting and then cultured the CD8^+^ T lymphocytes with cell-permeable S-2HG for 7 consecutive days to characterize the responses to the treatments. In the 200 μm S-2HG-treated cells, we observed an obvious increase in the percentages of Edu^+^ cells and effector CD8^+^ T lymphocytes compared with the control cells treated with PBS, suggesting that exogenous S-2HG promoted homeostatic proliferation (*F*_(2, 57)_ = 21.660, *P* < 0.0001) and differentiation (*F*_(2, 15)_ = 9.992, *P* = 0.0017) of CD8^+^ T lymphocytes (Fig. 6B, E). However, unexpectedly, 500 μm S-2HG treatment inhibited the homeostatic proliferation (*P* = 0.0014, vs. vehicle group) compared with control cells treated with PBS and produced only a trend toward decreasing in effector CD8^+^ T lymphocytes without statistical significance (*P* = 0.4406, vs. vehicle group) (Fig. 6B, E). Next, we sought to explore the role that endogenous S-2HG in CD8^+^ T lymphocyte function. L2hgdh is a crucial rate-limiting enzyme that can exert rapid oxidative degradation of S-2HG [26]. Compared to empty vector and PBS treated, transfection with lentiviral overexpression of L2hgdh (denoted as L2hgdh-Flag) into CD8^+^ T lymphocytes lead to the downregulation of Edu^+^ cells and effector CD8^+^ T lymphocytes, suggesting that endogenously produced S-2HG decreased proliferation (*F*_(2, 57)_ = 13.430, *P* < 0.0001) and triggered phenotypic markers conversion (*F*_(2, 12)_ = 6.364, *P* = 0.0131) (Fig. 6C, F). In contrast, knockdown of L2hgdh production with lentiviral shRNA (denoted as shL2hgdh) augmented proliferation (*F*_(2, 57)_ = 11.990, *P* < 0.0001) and differentiation (*F*_(2, 12)_ = 8.514, *P* = 0.0050) of CD8^+^ T lymphocytes compared with control shRNA (shScramble) and PBS (Fig. 6D, G). These results suggest that exogenous or endogenous S-2HG ex vivo regulates proliferation and phenotypic markers transformations of CD8^+^ T lymphocytes.

### S-2HG exacerbates the immune-mediated ischemic brain injury and cognitive dysfunction

**Fig. 7:**
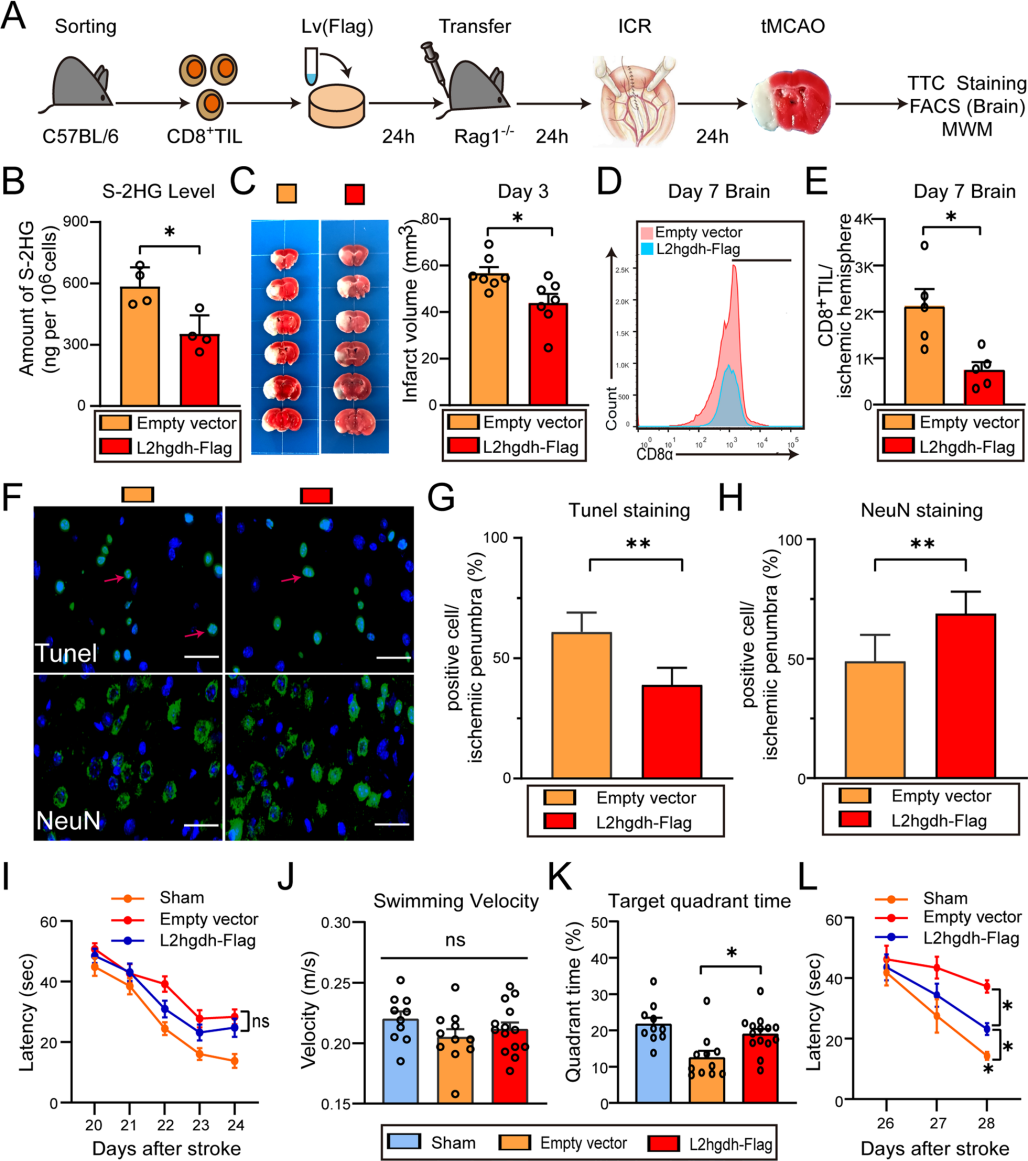
S-2HG exacerbates ischemic brain injury. **A** Flowchart illustrates the experimental design. **B** S-2HG levels in splenic CD8^+^ T cells transfected with lentiviral overexpression of L2hgdh. **C** Representative TTC-stained images and quantification (3 days after stroke) of Rag1^−/−^ mice with adoptively transferred CD8^+^ T cells transfecting with lentivirus L2ghdh-Flag (*n* = 7/group). **D**, **E** Representative histogram (**D**) and absolute number (**E**) of brain-invading CD8^+^ T cells (7 days after stroke) of Rag1^−/−^ mice with adoptively transferred CD8^+^ T cells transfecting with lentivirus L2ghdh-Flag (*n* = 5/group). **F** Representative images of brain tissue with Tunel staining or NeuN staining in the ischemic penumbra 7 days after stroke of Rag1^−/−^ mice with adoptively transferred CD8^+^ T cells transfecting with lentivirus L2ghdh-Flag. Red arrows signify the injured neurons. Scale bar = 20 μm. **G**, **H** Quantification of Tunel^+^ cells (**G**) or NeuN^+^ cells (**H**) (*n* = 5/group). **I** Average escape latency in the navigation test. **J** Quantification of swimming speed during the probe test. **K** Time traveled in the target quadrant during probe test (*n* = 10–14/group). **L** Quantification of latency to the platform during spatial working memory testing (*n* = 10–14/group). The sham mice were defined as the mice that underwent laparotomy without ICR. *P < 0.05, **P < 0.01, ns indicates nonsignificant

We further tested whether S-2HG exacerbated the ischemic brain injury and post-stroke cognition impairment by altering CD8^+^ T lymphocyte function (Fig. 7A). After isolating from healthy C57BL/6 mice spleen and transfecting with lentiviral overexpression of L2hgdh, we confirmed that S-2HG in CD8^+^ T cells decreased significantly (*t*_(6)_ = 3.559, *P* = 0.0119) by LC–MS (Fig. 7B). Next, 2 × 10^6^ transfected CD8^+^ T cells were injected via femoral vein to recipient Rag1^−/−^ mice before the perioperative stroke model established. Compared to Rag1^−/−^ mice with control cell transfer, a significant reduction in infarct volume were observed in Rag1^−/−^ mice with adoptively transferred CD8^+^ T cells at 3 days after tMCAO, indicating that L2hgdh activity upregulation and endogenous S-2HG reduction alleviate the ischemic brain injury (*t*_(12)_ = 2.738, *P* = 0.0180, Fig. 7C). Critically, great downregulation of brain-infiltrating CD8^+^ T lymphocytes was found in Rag1^−/−^ mice with adoptively transferred CD8^+^ T cells by the flow analysis of ischemic hemisphere compared to Rag1^−/−^ mice with control cell transfer, suggesting that a different redistribution of transferred CD8^+^ T cells occurred and less activated CD8^+^ T lymphocytes were recruited to the brain parenchyma (*t*_(8)_ = 3.226, *P* = 0.0121), which result in relatively decreased neurotoxic effect (Fig. 7D, E). Moreover, compared with Rag1^−/−^ mice with control cell transfer, reduced Tunel^+^ cells (*P* < 0.0001) and increased NeuN^+^ cells (*P* < 0.0001) in the ischemic penumbra were found in Rag1^−/−^ mice with adoptively transferred CD8^+^ T cells, suggesting the neuroprotection of L2hgdh activity upregulation (Fig. 7F, G).During the MWM test, no significant differences were detected in spatial learning ability (*P* = 0.0686, Fig. 7I) and swimming velocity in the probe test (*F*_(2, 32)_ = 1.417, *P* = 0.2572, Fig. 7J). Rag1^−/−^ mice with adoptively transferred CD8^+^ T cells had a reduction of deficits in spatial reference memory (*F*_(2, 32)_ = 7.947, *P* = 0.0016, Fig. 7K) and spatial working memory (*P* = 0.0186, Fig. 7L) than Rag1^−/−^ mice with control cell transfer, suggesting that endogenous S-2HG reduction alleviated post-stroke cognitive dysfunction.

## Discussion

Perioperative ischemic stroke is an under-appreciated and catastrophic neurological complication of surgery with high mortality and disability [27]. Nevertheless, the mechanisms by which perioperative stroke occurs remain largely uncertain. In the present study, we are focused on the integration effects of perioperative risk factors such as surgical insults, traumatic injuries, anesthesia, or hypoxia and demonstrated that the immunometabolite S-2-hydroxyglutarate exacerbated perioperative ischemic brain injury and post-stroke cognitive dysfunction with structural and functional brain changes by enhancing CD8^+^ T lymphocyte-mediated neurotoxicity. The potential clinical implication of our findings is that S-2HG and CD8^+^ T lymphocyte could become the promising immune-modulatory targets for perioperative ischemic stroke therapy.

The blood–brain barrier protects the brain from most endogenous and exogenous risk factors. However, neuroinflammation in CNS disorders can potentially disrupt BBB structural integrity and increase BBB permeability, while even peripheral systemic inflammation trigger BBB damage [28]. During the ischemic stroke event, substantial inflammatory factors or cytokines released from necrotic neurons enter the peripheral circulation through the pathological BBB, which may affect the activation of peripheral immune cells that do not enter the brain parenchyma during physiological state [29]. Moreover, migration and adhesion of activated peripheral immune cells to disrupted BBB and brain parenchyma will further exacerbate the BBB damage and ischemic brain injury [30]. In our study, we demonstrated that tight junction protein occludin degradation and BBB disruption were progressively worse with the potentially augmenting inflammation response in perioperative stroke mice. We reasoned that the dual origin of inflammatory factors from both surgical intervention and necrotic neuronal death induced exacerbated BBB disruption in perioperative stroke mice. We went a step further and focused on the role of immune responses and secondary neuronal damage in perioperative ischemic brain injury. We observed that the invasion of brain parenchyma and direct neurotoxicity of CD8^+^ T lymphocytes may play a crucial role in ischemic brain injury and contribute to the exacerbation of ischemic brain injury in perioperative stroke mice. The deleterious effects of CD8^+^ T lymphocytes are more mediated via direct cytotoxicity rather than humoral pathways in perioperative ischemia. Furthermore, we performed the experimental paradigm of CD8^+^ T lymphocytes depletion using antibody neutralization that confirmed the deleterious effect in immune-mediated cerebral ischemic brain injury of CD8^+^ T lymphocytes in perioperative stroke mice.

In response to T cell receptors (TCRs) triggering, quiescent CD8^+^ T-lymphocytes convert to memory CD8^+^ T cells and effector T cells [31–33]. Cellular metabolism controls CD8^+^ T cells activation and differentiation. The metabolic programs of CD8^+^ T lymphocyte states are important for the expression of key phenotypic markers [34]. To systematically identify metabolic factors that induce the activation and differentiation of CD8^+^ T lymphocytes, we performed untargeted metabolomics on sorted splenic CD8^+^ T lymphocytes from the stroke models and demonstrated that the differential metabolites in perioperative stroke mice were mainly enriched in early citrate cycle (TCA cycle) metabolism, which is associated with glucose utilization and oxygen consumption [35]. These results indicated that the CD8^+^ T lymphocytes of perioperative stroke mice underwent a massive metabolic switch to adapt to demands of cell growth and differentiation due to the integration effects of perioperative risk factors such as surgical insults, traumatic injuries, anesthesia, or hypoxia.

**Fig. 8:**
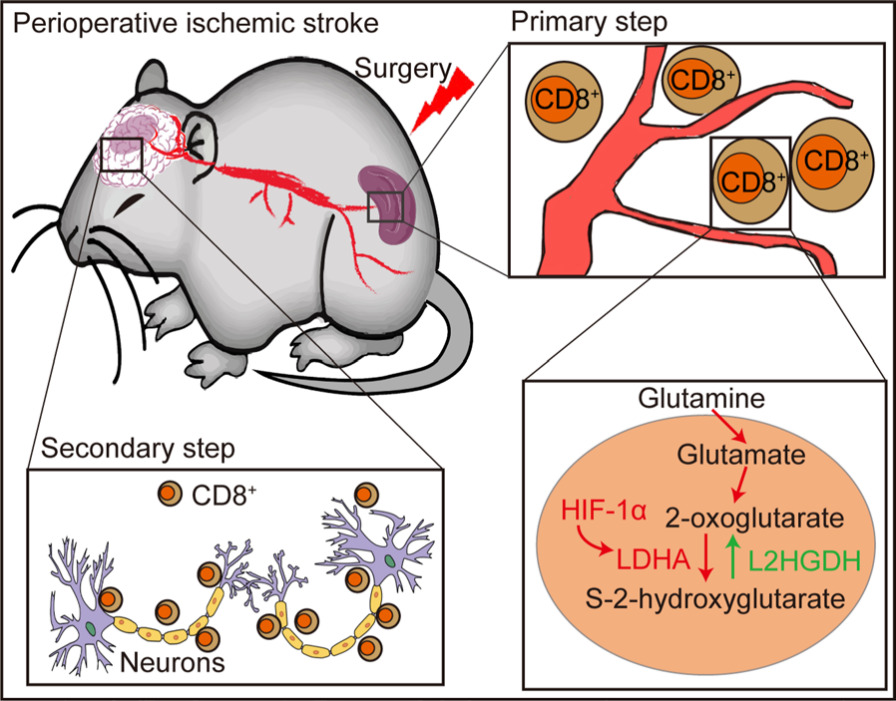
Schematic illustrating novel mechanism underlying S-2HG-mediated perioperative ischemic brain injury. Perioperative risk factors trigger an aberrant metabolic accumulation of S-2HG in peripheral CD8^+^ T cells and thus enhance the activation of CD8^+^ T cells. During the ischemic stroke event, migration and adhesion of activated peripheral CD8^+^ T cells to disrupted BBB and brain parenchyma will further exacerbate ischemic brain injury and neurological dysfunction

S-2-Hydroxyglutarate is present in urine of healthy individuals and is elevated in patients with an inborn metabolic disease that results from L2hgdh deficiency [36]. Emerging studies highlight that accumulation of S-2HG can modulate the differentiation of CD8^+^ T lymphocytes through altering histone and DNA demethylation and thus trigger the immune response [10, 37]. The productions of S-2HG are derived from lactate dehydrogenase or malate dehydrogenase and are strongly dependent on HIF-1α signaling [38, 39]. Furthermore, the expression of lactate dehydrogenase in hypoxic environment is HIF-1α-dependent and makes a greater contribution to hypoxia-induced S-2HG production than the production of malate dehydrogenase. Consistent with these previous studies, we also found that HIF-1α and LDHA, triggered by inflammatory and hypoxic responses, strikingly increased in perioperative stroke mice compared with stroke-only mice and sham mice. We observed that the accumulation of S-2HG in CD8^+^ T cells in response to TCR triggering and hypoxia significantly increased in perioperative stroke mice compared to stroke-only mice. In a normal physiological state, the production of S-2HG relies heavily on oxidative phosphorylation and involves glucose, acetyl-CoA, and α-oxoglutarate. However, in the context of hypoxia and mitochondrial deficit, glutamine is the major source of S-2HG with lower glucose and oxygen consumption [40]. In addition, we used the treatment with cell-permeable S-2HG and the modulation of L2hdgh activity with lentiviral transfection to alter the S-2HG levels and demonstrated that exogenous or endogenous S-2HG ex vivo regulates proliferation and differentiation of CD8^+^ T lymphocytes. Nevertheless, when 500 μm S-2HG treatment with CD8^+^ T lymphocyte inhibited the homeostatic proliferation compared with control cells, we reasoned that there may be an increase in apoptosis at the dose due to drug-related toxicities. Using selective adoptive CD8^+^ T cells transfer into Rag1^−/−^ mice in vivo, endogenous S-2HG reduction alleviates the ischemic brain injury and post-stroke cognitive dysfunction with fewer brain-infiltrating CD8^+^ T lymphocytes. These data support our conclusion that immunometabolite S-2HG may exacerbate perioperative ischemic brain injury and post-stroke cognitive dysfunction (Fig. 8).

However, in the current study, we did not evaluate the influence of adoptive CD8^+^ T cells transfected with lentiviral L2hgdh shRNA transfer into Rag1^−/−^ mice in vivo on ischemic brain injury and MWM, which warrants further attention. Given the neuroprotection with female hormones and the appropriate number of animals to reach statistical significance, we only chose male mice in this study.

## Conclusion

Our study advances an important new understanding of the mechanisms of immune-mediated ischemic brain injury and post-stroke cognitive dysfunction with perioperative ischemic stroke. Invasion of brain parenchyma and direct neurotoxicity of CD8^+^ T lymphocytes may play a critical role in immune-mediated ischemic brain injury. Aberrant accumulation of immunometabolite S-2HG mediates activation and migration of CD8^+^ T lymphocytes, which may present a novel mechanism and therapeutic target underlying exacerbation effect of perioperative ischemic stroke.

## Supplementary Information

**Additional file 1: Figure S1.** The perioperative stroke mice exhibit more severe ischemia-induced neuronal injury in the penumbra. **A** Representative images of brain tissue with HE staining (a), Nissl staining (b), Tunel staining (c) or NeuN staining (d) in the ischemic penumbra 7 days after stroke. Black or white arrows denote the intact neurons with flush cell bodies. Red arrows signify the injured neurons. Scale bar = 20 μm. **B**–**E** Quantification of HE^+^ cells (complete cells) (**B**), Nissl^+^ cells (intact neurons) (**C**), Tunel^+^ cells (apoptotic neurons) (**D**) or NeuN^+^ cells (intact neurons) (**E**) (*n* = 5/group). *P < 0.05, **P < 0.01. **Figure S2.** The perioperative stroke mice are more vulnerable to develop profound sensorimotor impairments. **A**–**F** Sensorimotor function was assessed using Garcia score test including body proprioception (**A**), climbing (**B**), forelimb walking (**C**), limb symmetry (**D**), lateral turning (**E**), and total neurological score (**F**) (*n* = 10–15/group). *P < 0.05, **P < 0.01. **Figure S3.** Similar brain-infiltrating CD4^+^ T lymphocyte, B cell, or neutrophil response in perioperative stroke and stroke-only mice. **A**–**F** Representative dot plots and quantifications of CD4^+^ T cells (**A**, **B**), B cell (**C**, **D**) and neutrophil (**E**,** F**) in blood, spleen or brain 7 days after stroke (*n* = 5–6/group). *P < 0.05, **P < 0.01, ns indicates nonsignificant. **Figure S4.** Brain invasion of CD8^+^ T lymphocytes exacerbates ischemic brain injury in perioperative stroke mice. **A** Representative immunostaining of CD8^+^ T cells, CD4^+^ T cells, GFAP^+^ astrocytes, or Iba1^+^ microglia of mice brain sections. Scale bars = 20 μm. **B**, **C** Quantification of brain infiltrating CD8^+^ T cells (**B**) or CD4^+^ T cells (**C**) in ischemic hemisphere by immunofluorescence in 5 consecutive coronal sections (1 mm apart) (*n* = 5/group). **D** Quantification of the average area of GFAP^+^ astrocyte or Iba1^+^ microglia in ischemic penumbra by immunofluorescence in 5 consecutive coronal sections (1 mm apart) (*n* = 5/group). *P < 0.05, **P < 0.01, ns indicates nonsignificant.

## Data Availability

The datasets during and/or analyzed during the current study are available from the corresponding author on reasonable request.

## Abbreviations

S-2HG: S-2-Hydroxyglutarate
Rag1: Recombination activating gene 1
ICR: Ileocecal resection
MCA: Middle cerebral artery
TR: Repetition time
TE: Echo time
FOV: Field of view
L2hgdh: L-2-Hydroxyglutarate dehydrogenase
MWM: Morris water maze
HIF-1α: Hypoxia-inducible factor 1alpha
LDHA: Lactate dehydrogenase A
TTC: 2,3,5-triphenyltetrazolium chloride
MAP2: Microtubule-associated protein 2
HE: Hematoxylin and eosin
tMCAO: Transient middle cerebral artery occlusion
BBB: Blood–brain barrier
FCM: Flow cytometry
mAb: Monoclonal antibodies
PLS-DA: Partial least-squares discrimination analysis
GFAP: Glial fibrillary acidic protein
Edu: 5-Ethynyl-2′-deoxyuridine
