# Supplementary material for: The immunometabolite S-2-hydroxyglutarate exacerbates perioperative ischemic brain injury and cognitive dysfunction by enhancing CD8+ T lymphocyte-mediated neurotoxicity
Source: J Neuroinflammation. 2022 Jul 7;19:176. doi: 10.1186/s12974-022-02537-4 (PMC9264651; doi:10.1186/s12974-022-02537-4)
Supplement: Supplementary file 1 — Additional file 1: Figure S1. The perioperative stroke mice exhibit more severe ischemia-induced neuronal injury in the penumbra. A Representative images of brain tissue with HE staining (a), Nissl staining (b), Tunel staining (c) or NeuN staining (d) in the ischemic penumbra 7 days after stroke. Black or white arrows denote the intact neurons with flush cell bodies. Red arrows signify the injured neurons. Scale bar = 20 μm. B–E Quantification of HE+ cells (complete cells) (B), Nissl+ cells (intact neurons) (C), Tunel+ cells (apoptotic neurons) (D) or NeuN+ cells (intact neurons) (E) (n = 5/group). *P < 0.05, **P < 0.01. Figure S2. The perioperative stroke mice are more vulnerable to develop profound sensorimotor impairments. A–F Sensorimotor function was assessed using Garcia score test including body proprioception (A), climbing (B), forelimb walking (C), limb symmetry (D), lateral turning (E), and total neurological score (F) (n = 10–15/group). *P < 0.05, **P < 0.01. Figure S3. Similar brain-infiltrating CD4+ T lymphocyte, B cell, or neutrophil response in perioperative stroke and stroke-only mice. A–F Representative dot plots and quantifications of CD4+ T cells (A, B), B cell (C, D) and neutrophil (E, F) in blood, spleen or brain 7 days after stroke (n = 5–6/group). *P < 0.05, **P < 0.01, ns indicates nonsignificant. Figure S4. Brain invasion of CD8+ T lymphocytes exacerbates ischemic brain injury in perioperative stroke mice. A Representative immunostaining of CD8+ T cells, CD4+ T cells, GFAP+ astrocytes, or Iba1+ microglia of mice brain sections. Scale bars = 20 μm. B, C Quantification of brain infiltrating CD8+ T cells (B) or CD4+ T cells (C) in ischemic hemisphere by immunofluorescence in 5 consecutive coronal sections (1 mm apart) (n = 5/group). D Quantification of the average area of GFAP+ astrocyte or Iba1+ microglia in ischemic penumbra by immunofluorescence in 5 consecutive coronal sections (1 mm apart) (n = 5/group). *P < 0.05, **P < 0.01, ns indicates [file 12974_2022_2537_MOESM1_ESM.docx]

**
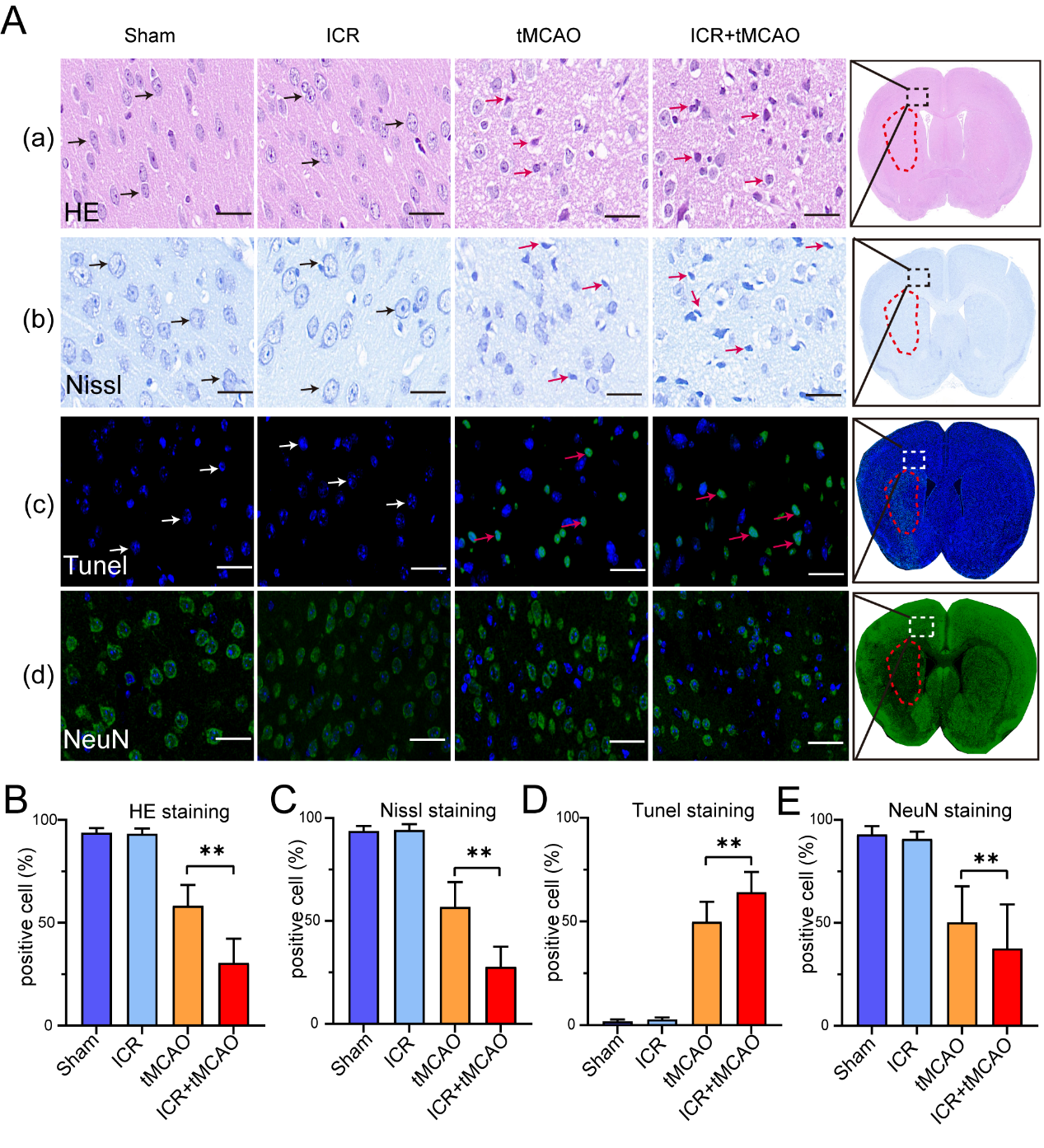
 Figure S1 The perioperative stroke mice exhibit more severe ischemia-induced neuronal injury in the penumbra**. **A** Representative images of brain tissue with HE staining (a), Nissl staining (b), Tunel staining (c) or NeuN staining (d) in the ischemic penumbra 7 days after stroke. Black or white arrows denote the intact neurons with flush cell bodies. Red arrows signify the injured neurons. Scale bar =20 μm. **B-E** Quantification of HE^+^ cells (complete cells) (**B**), Nissl^+^ cells (intact neurons) (**C**), Tunel^+^ cells (apoptotic neurons) (**D**) or NeuN^+^ cells (intact neurons) (**E**) (n = 5/group). **P* < 0.05, ***P* < 0.01.


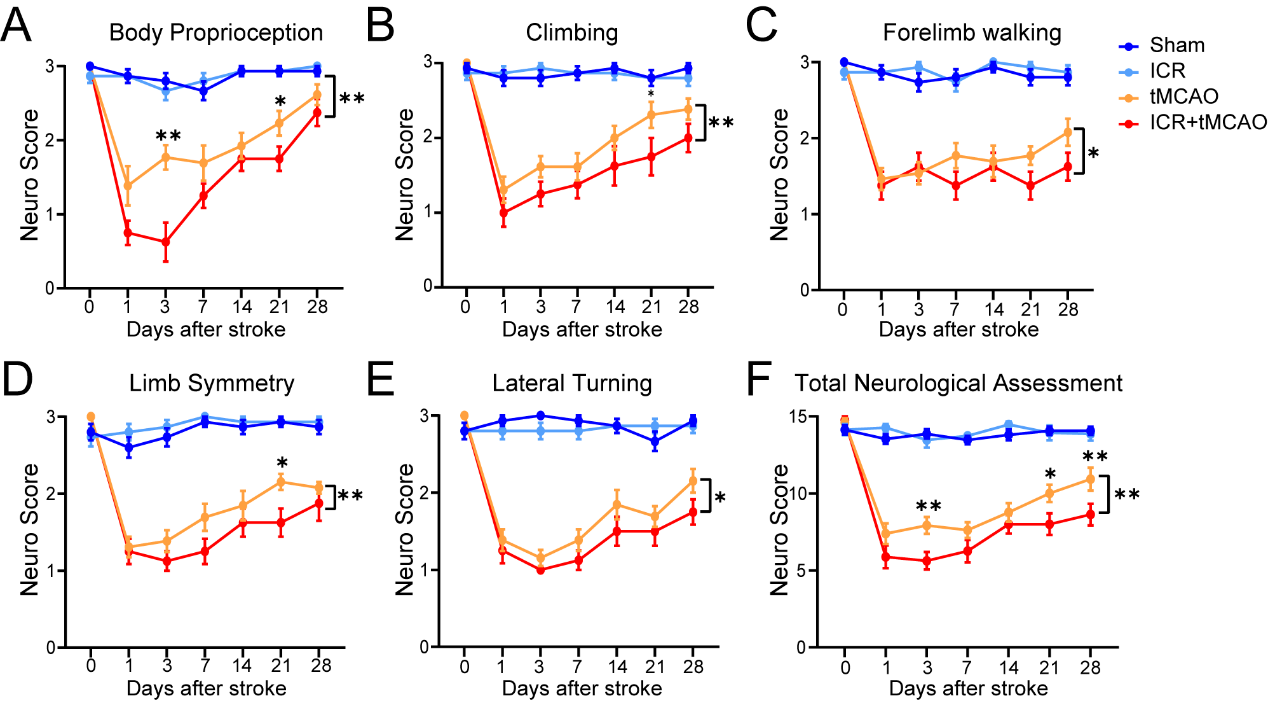


**Figure S2 The perioperative stroke mice are more vulnerable to develop profound sensorimotor impairments. A-F** Sensorimotor function was assessed using Garcia score test including body proprioception (**A**), climbing (**B**), forelimb walking (**C**), limb symmetry (**D**), lateral turning (**E**), and total neurological score (**F**) (n = 10-15/group). **P* < 0.05, ***P* < 0.01.


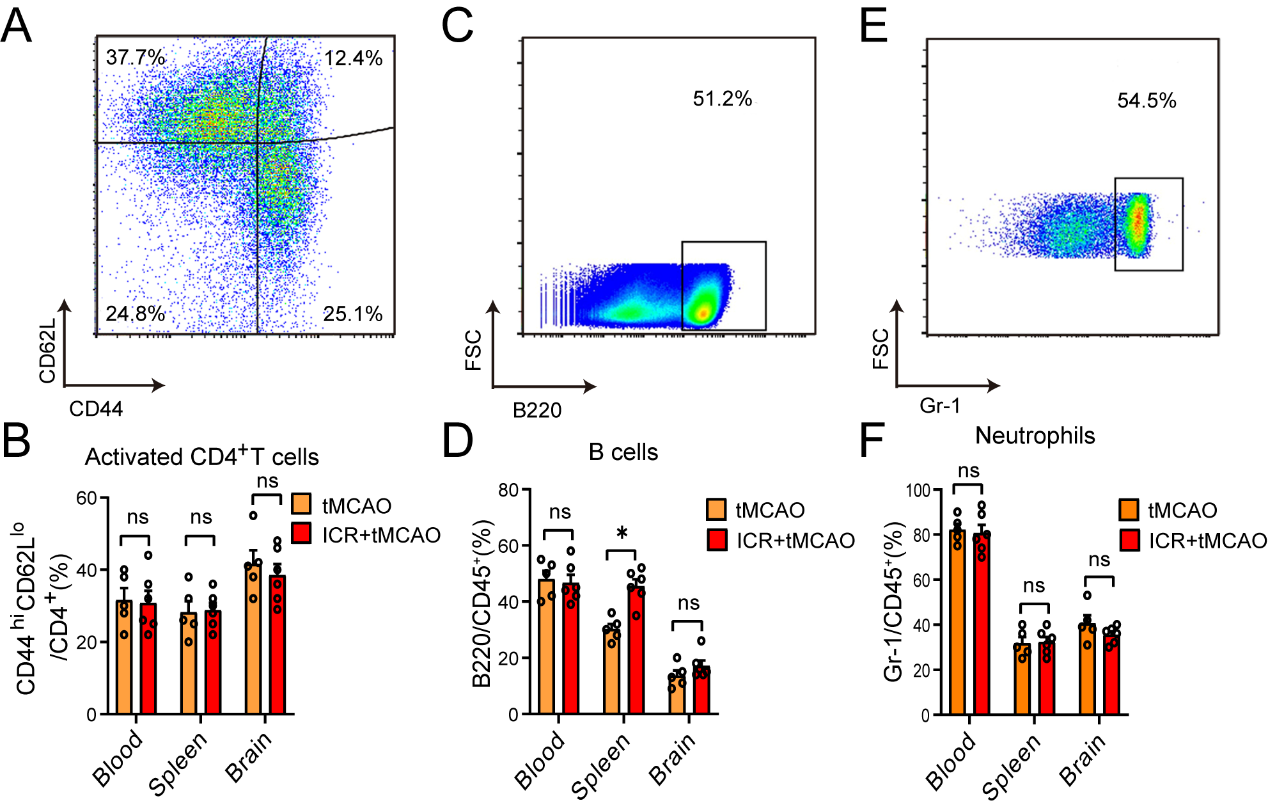
 **Figure S3 Similar brain-infiltrating CD4^+^ T lymphocyte, B cell, or neutrophil response in perioperative stroke and stroke-only mice.** **A-F** Representative dot plots and quantifications of CD4^+^ T cells (**A, B**), B cell (**C, D**) and neutrophil (**E, F**) in blood, spleen or brain 7 days after stroke (n = 5-6/group). **P* < 0.05, ***P* < 0.01, ns indicates nonsignificant.


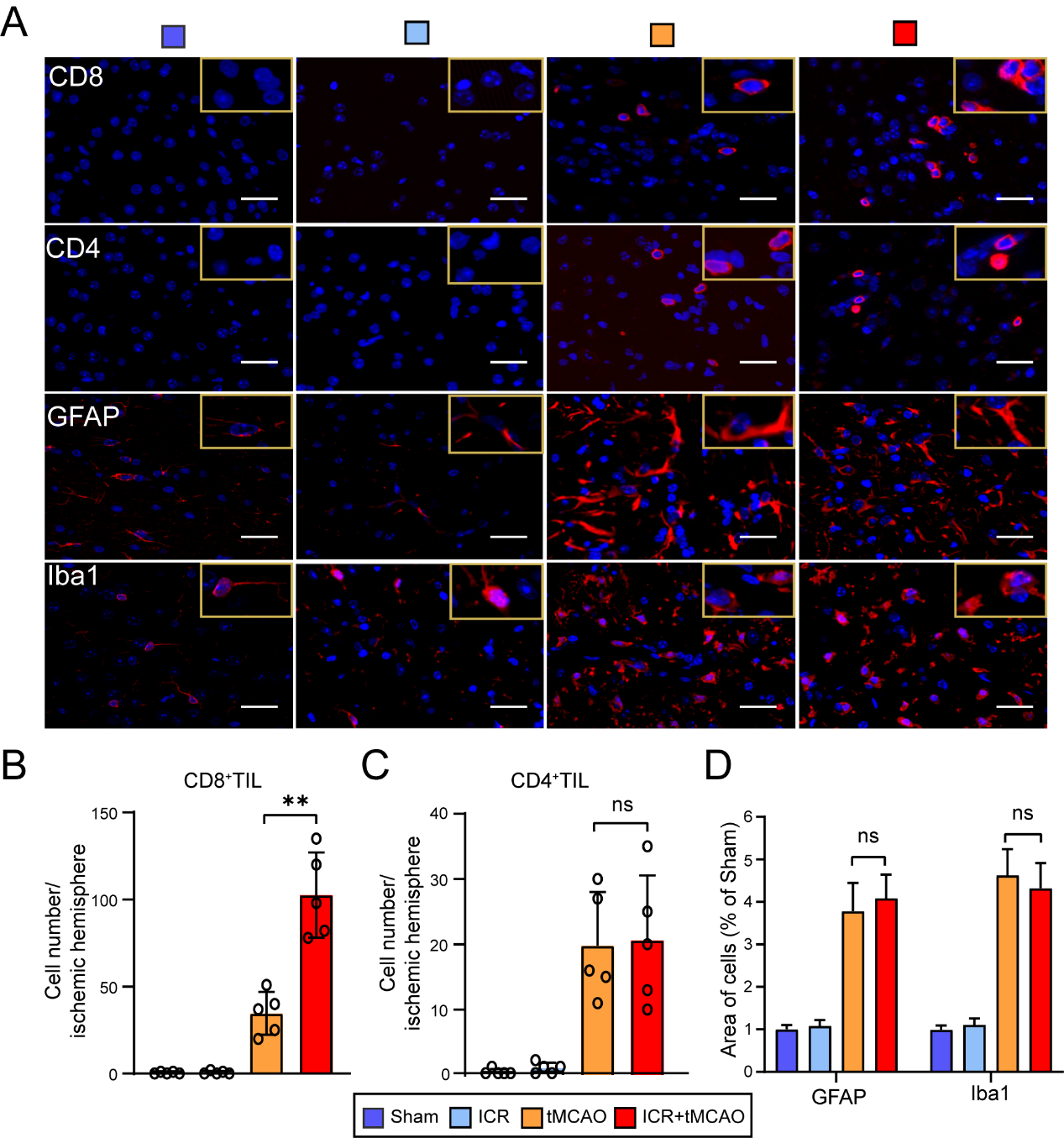


**Figure S4 Brain invasion of CD8^+^ T lymphocytes exacerbates ischemic brain injury in perioperative stroke mice. A** Representative immunostaining of CD8^+^ T cells, CD4^+^ T cells, GFAP^+^ astrocytes, or Iba1^+^ microglia of mice brain sections. Scale bars = 20 μm. **B, C** Quantification of brain infiltrating CD8^+^ T cells **(B)** or CD4^+^ T cells **(C)** in ischemic hemisphere by immunofluorescence in 5 consecutive coronal sections (1mm apart) (n = 5/group). **D** Quantification of the average area of GFAP^+^ astrocyte or Iba1^+^ microglia in ischemic penumbra by immunofluorescence in 5 consecutive coronal sections (1mm apart) (n = 5/group). **P* < 0.05, ***P* < 0.01, ns indicates nonsignificant.
